# A Pediatric Case of Ketosis-Prone Type 2 Diabetes Requiring Insulin Therapy

**DOI:** 10.7759/cureus.23305

**Published:** 2022-03-18

**Authors:** Misa Matsuura, Daisuke Sugawara, Ko Ichihashi

**Affiliations:** 1 Pediatrics, Saitama Medical Center, Jichi Medical University, Saitama, JPN

**Keywords:** ketosis-prone diabetes, pediatric diabetes, insulin treatment, pediatric weight management, diabetic ketoacidosis (dka)

## Abstract

In recent years, cases of diabetic ketoacidosis (DKA) or ketosis as the initial manifestation of diabetes with a similar clinical course to that of type 2 diabetes have been reported. This phenotype has been recognized as ketosis-prone type 2 diabetes (KPD). Most cases of KPD occur in adults, and the typical clinical course is that patients are often able to wean off insulin therapy after initial treatment. We report a pediatric case of ketosis-prone type 2 diabetes requiring insulin therapy with four years of follow-up. Our case suggests that patients with KPD may require strict management, including weight control, compared with patients with typical type 2 diabetes.

## Introduction

It is assumed that most patients presenting with diabetic ketoacidosis (DKA) or ketosis have type 1 diabetes. However, some patients subsequently develop clinical and metabolic features of type 2 diabetes despite presenting with DKA. This clinical presentation is recognized as ketosis-prone type 2 diabetes (KPD) [1]. Most cases of KPD occur in adults, and the typical clinical course is that patients are often able to wean off insulin therapy after initial treatment [2]. Little has been reported on KPD in children, and the clinical course of childhood-onset KPD is not well understood [3,4]. We report a pediatric case of ketosis-prone type 2 diabetes requiring insulin therapy with four years of follow-up.

## Case presentation

A 12-year-old male experienced polydipsia for one month along with fatigue, nausea, and poor appetite that had persisted for several days. He also experienced rapid weight loss from 75.6 kg (BMI: 25.6 kg/m^2^) to 65.4 kg within three weeks. He was admitted to our hospital with a complaint of a stomachache. On admission, he had a temperature of 98.2°F, blood pressure of 100/68 mmHg, a pulse of 91 beats per minute, and oxygen saturation of 100% on room air. On physical examination, consciousness is clear, and chest and abdomen findings were unremarkable. There was acanthosis nigricans in his armpits and neck. He had a family history of diabetes. His mother had a history of gestational diabetes, and his older stepbrother was diagnosed with type 2 diabetes at 21 years of age. His initial laboratory data on admission are shown in Table 1.

**Table 1:** Laboratory data obtained on admission

Laboratory test	Value	Reference
Complete blood count		
White blood cell count	12,610/µL	4,000–10,700/µL
Hemoglobin	18.7 g/dL	12.2–15.7 g/dL
Hematocrit	56%	35.8%–48%
Platelets	32.6 × 10^4^/µL	18–44 × 10^4^/µL
Blood chemistry		
Total protein	8.3 g/dL	6.3–7.8 g/dL
Albumin	5.2 g/dL	3.8–4.7 g/dL
Aspartate aminotransferase	8 IU/L	15–31 IU/L
Alanine aminotransferase	7 IU/L	9–32 IU/L
Amylase	243 IU/L	37–120 IU/L
Blood urea nitrogen	19 mg/dL	6.8–19.2 mg/dL
Creatinine	0.68 mg/dL	0.4–0.81 mg/dL
Sodium	129 mEq/L	138–144 mEq/L
Potassium	5 mEq/L	3.6–4.7 mEq/L
Chloride	97 mEq/L	102–109 mEq/L
Calcium	8.9 mg/dL	8.7–10.1 mg/dL
Phosphorus	5.1 mg/dL	3.6–5.8 mg/dL
C-reactive protein	0.11 mg/dL	<0.3 mg/dL
Total ketone body	12,612 µmol/L	<130 µmol/L
Acetoacetic acid	2,286 µmol/L	<55 µmol/L
3-Hydroxybutyric acid	10,326 µmol/L	<85 µmol/L
Glycated hemoglobin	12.8%	4.6%–6.2%
C-peptide	1.21 ng/mL	>0.6 ng/mL
Glucose	694 mg/dL	70–140 mg/dL
Blood gas analysis (venous)		
pH	7.086	7.35–7.45
Carbon dioxide partial pressure	16.1 mmHg	35–45 mmHg
Bicarbonate	4.7 mmol/L	23–28 mmol/L
Base excess	-22.7 mmol/L	-2.2–+1.2 mmol/L
Anion gap	27.3 mEq/L	12 ± 2 mEq/L

Blood glucose level was 694 mg/dL, total ketone body was 12.6 mmol/L, and glycated hemoglobin (HbA1c) level was 12.8%. Venous blood gas analysis showed the following findings: pH, 7.086; pCO2, 16.1 mmHg; bicarbonate, 4.7 mEq/L; and base excess, -22.7 mEq/L.

Based on these results, despite the presence of severe acidosis and hyperketonemia, blood glucose was markedly high and hyperosmotic. Therefore, he was suspected of a mixture of DKA and hyperosmolar hyperglycemic syndrome. The patient was initially treated with fluid infusion (0.9% saline 500 mL/hour). After starting fluid replacement therapy, continuous intravenous insulin therapy (insulin human: 0.05 unit/kg) and 30 mEq of potassium repletion were started. He responded well to these initial treatments, and his treatment was changed to subcutaneous insulin therapy. Short-acting insulin (glargine) and long-acting insulin (degludec) were used for subcutaneous insulin infusions. The total daily dose of insulin was 1 unit/kg. He learned the technique of insulin injection and was discharged on the 22nd day of hospitalization. Glucotoxicity improved after treatment, and a glucagon stimulation test (1 mg of glucagon intravenous infusion early in the morning on an empty stomach) was performed at the time of discharge (Table 2).

**Table 2:** Result of glucagon stimulation test

Variable	Value
C-peptide at baseline	0.92 ng/mL
C-peptide at five minutes after stimulation	3 ng/mL
C-peptide at 10 minutes after stimulation	2.81 ng/mL

The fasting C-peptide level was 0.92 ng/mL (normal: >0.6 ng/mL),and the peak serum C-peptide level was 3 ng/mL (normal: >2 ng/mL). These results suggest that β-cell function was preserved, contrary to the expected findings in acute-onset type 1 diabetes.

After discharge, his serum C-peptide level increased to 8.96 ng/mL, and HbA1c decreased to 6%. At one month after discharge, he started metformin (1500 mg/day) in addition to insulin therapy. His insulin dose was then progressively reduced to 0.5 units/kg and eventually discontinued six months after the initial DKA episode. He continued to visit the hospital once a month, and we checked his compliance with oral administration of metformin and blood glucose monitoring three times a day. However, 12 months after the diagnosis, his weight had gradually increased by a total of 14 kg, and his HbA1c level had increased to 7.9%. At this time, he developed ketosis again, and his serum C-peptide level decreased to 2.12 ng/mL, at which point insulin therapy was restarted. The patient’s glycemic control gradually improved after resuming the insulin therapy (HbA1c: 7.2%). However, his serum C-peptide level remained lower than previously recorded levels (1.67 ng/mL). He resumed insulin therapy 12 months after the onset of DKA and has continued insulin therapy ever since. Four years after the first episode of DKA, he experienced three episodes of ketosis.

We have listed type 1 diabetes mellitus, type 2 diabetes mellitus, maturity-onset diabetes of the young (MODY), and KPD as differential diagnoses and conducted further examinations. No islet-cell antibodies (autoantibodies against glutamic acid decarboxylase, islet antigen 2 antibodies, anti-insulin antibodies, or antibodies against zinc transporter 8) were detected. His serum C-peptide level was 4.89 ng/mL, and his residual insulin secretion was still maintained. Exome sequencing of *MODY 1-14* genes (*HNF4A*,* GCK*,* HNF1A*,* PDX1*,* HNF1B*,* NEUROD1*,* KLF11*,* CEL*,* INS*,* BLK*,* ABCC8*,* KCNJ11*, and* APPL1*) was analyzed in the patient and his brother, and no mutations were detected. Human leukocyte antigen (HLA) class II genotyping was performed with polymerase chain reaction-based sequence-specific primers. The results showed that his HLA class II haplotype was DRB1*0405-DQB1*0302. Although his clinical course was comparable to that of type 2 diabetes, no triggers for DKA, such as the consumption of large amounts of soft drinks, infection, operation, or injury, were observed. Moreover, the patient had repeated episodes of diabetic ketosis. These clinical courses are similar to those of KPD, and he was diagnosed with KPD.

## Discussion

We report a pediatric case of KPD who experienced persistent insulin therapy four years after his initial diagnosis. Although there are no strict diagnostic criteria for KPD, the clinical features are as follows [5-7]: (1) ketosis or ketoacidosis developing without any triggers, polydipsia polyuria, and loss of body weight occurring within 4-6 weeks of diagnosis; (2) more than 80% of patients have a family history of type 2 diabetes mellitus; (3) most patients newly diagnosed with KPD present with obesity; and (4) the clinical course is such that insulin therapy can be discontinued early after the initial treatment, but hyperglycemia and ketosis recur frequently within a few years.

KPD is categorized based on the presence or absence of islet-cell autoantibodies (A+, A-) and β-cell functional reserve (β+, β-). This classification is called the Aβ system [8]. In our case, the patient was categorized in the A-β+group. Patients in the A-β+ group often do not need insulin therapy after initial treatment [9]. However, he still required ongoing insulin therapy four years from the onset of KPD. Compared with A-β+ patients whose β-cell function is preserved, A-β+ patients whose β-cell function gradually declines have a higher prevalence of the HLA class II allele DQB1*0302 [10]. The HLA haplotype of the patient was found to be DRB1*0405-DQB1*0302, and this may be related to the patient requiring insulin therapy.

In some cases of KPD, weight gain is related to the recurrence of ketosis and results in persistent insulin therapy [11]. It is speculated that this occurs because of β-cell overload due to the increased insulin resistance caused by body weight gain, which impairs insulin secretion and results in ketosis. In our case, the patient had three episodes of ketosis after onset, all of which were seen after weight gain.

## Conclusions

Our report suggests that there are some patients who continue insulin therapy, and recurrent ketosis may be related to body weight gain in the pediatric population. Therefore, patients with KPD may require strict management, including weight control, compared with patients with typical type 2 diabetes.
